# The bumpy road to change: a retrospective qualitative study on formerly detained adolescents’ trajectories towards better lives

**DOI:** 10.1186/s13034-019-0271-6

**Published:** 2019-02-25

**Authors:** Nele Van Hecke, Wouter Vanderplasschen, Lore Van Damme, Stijn Vandevelde

**Affiliations:** Department of Special Needs Education, Ghent University, Henri Dunantlaan 2, 9000 Ghent, Belgium

**Keywords:** Longitudinal studies, Young offenders, Quality of life, Good Lives Model, Rehabilitation, Qualitative studies

## Abstract

**Background:**

Currently, the risk-oriented focus in forensic youth care is increasingly complemented by a growing interest in strengths-based approaches. Knowledge on how detention and the subsequent period in the community is experienced by adolescents, and which elements are helpful in achieving better lives can contribute to this emerging field. The current study aimed to retrospectively explore adolescents’ experiences from the moment they were detained until 6 to 12 months after they left the institution, identifying positive aspects and strengths.

**Methods:**

In-depth interviews were conducted with 25 adolescents (both boys and girls, 15–18 years old) on average 8 months after discharge from a closed institution in Belgium. A thematic analysis was performed using NVivo 11.

**Results:**

Five themes and corresponding subthemes were identified: (1) adolescents’ own strengths and resilience, (2) re-building personally valued lives, (3) making sense of past experiences, (4) moving away from a harmful lifestyle, and (5) (in-)formal supports. Most adolescents are on their way to finding a new balance in life, however, for a subgroup of them, this is still fragile. Adolescents highly emphasize the importance of feeling closely connected to at least one person; to receive practical help with regard to finances, work and housing; and to be able to experience pleasure and joy in their lives.

**Conclusions:**

Adolescents’ narratives suggest that starting a journey towards a normative good life often goes along with an initial difficult period because of a sense of loss with regard to their former life. This stresses the importance of targeting rehabilitation towards prosocial goals and enhancing adolescents’ quality of life on those life domains that matter most for them. Furthermore, we stress the importance of helping adolescents in overcoming structural barriers as a first step in supporting them in their trajectories towards better lives.

## Background

Research and practice in the field of forensic youth care have traditionally been characterized by a problem-oriented approach and a predominant focus on reducing the risk of reoffending [1, 2]. In recent years, this has been complemented with strengths-based approaches, focusing on both offenders’ risks and needs, as well as their wellbeing and capacities [3, 4]. The Good Lives Model of Offender Rehabilitation (GLM) [5, 6] is a holistic strengths-based approach in the field of correctional services and forensic care. The GLM is a theoretical rehabilitation framework originally developed for adult offenders [7], that has recently been studied and theoretically discussed in relation to adolescent populations as well [2, 5, 6].

The GLM encompasses a dual focus on both enhancing offenders’ wellbeing, while at the same time reducing their risk of re-offending [4]. Supporting offenders in pursuing their goals is, from a GLM point of view, inextricably entangled with motivating them towards leading a ‘good life’—a personally valuable and meaningful life, within the contours of what is socially acceptable [4, 7]. However, in the group of adolescents who have been ‘detained’, little is known about what they perceive as personally valuable and meaningful. Listening to the stories and experiences of detained adolescents may provide us with a better understanding about what supports them in their desistance process, but also—and maybe even more importantly—inform us more broadly on what is meaningful to them, and what contributes to the acquisition and development of a good (quality of) life [3]. The present study aims to highlight adolescents’ experiences, with a focus on positive aspects and strengths, on their way to ‘better’ lives—both from a personal and normative point of view. As such, we combine the focus of desistance research on socially desirable outcomes, with a more client-centered perspective, focusing on quality of life. In this study, we retrospectively shed light on adolescents’ experiences from the moment they were ‘detained’ until 6 to 12 months after they left the closed institution for mandatory care and treatment (CI).^1^ Furthermore, we aim to investigate how and to what extent this period in the CI influenced their trajectories towards change.

The focus of our study is situated at the intersection between several closely related, but nonetheless distinct strengths-based concepts such as recovery, inclusion and desistance. The common denominator of these concepts is that they all imply a gradual change/shift from one situation to another, more desirable situation; which takes place in and affects different areas of one’s life. We choose not to set out specific criteria to predefine change, but rather to operationalize it as a certain form of ‘improvement’ or ‘sense of progress in life’ [8] as perceived and experienced by adolescents themselves in their daily lives and in relation to their context and the broader society. This is in accordance with Vandevelde and colleagues [3] who—building on the integrative stance of Broekaert and colleagues [9]—suggest an understanding of ‘improvement’ by “the dialectical transaction/dialogue between all actors in their daily interactions […] for each and every individual” (p. 77). As such, any notion of change in the sense of improvement—although individually perceived—cannot be detached from a broader societal and normative framework, with its own expectations and conceptions of what constitutes ‘good’ and acceptable behavior. This balance between guiding people towards ‘better’ lives, within normative boundaries, is at the heart of the GLM [4, 7], and is particularly salient with regard to adolescents. Notwithstanding that most individuals cope successfully with the developmental demands connected to adolescence, this period is typically characterized by elevated levels of turmoil [10], especially in relation to mood disturbances, increased risk taking and conflict [11]. Adolescence can be seen as a period in which relational and normative boundaries are explored, probed and sometimes crossed, in an attempt to position oneself in relation to others and society, and in the process of discovering and developing one’s own identity. Furthermore, adolescents are particularly susceptible to environmental influences, characterized by a gradually increasing importance of friends and decreasing importance of parents [6].

Studies investigating adolescents’ perception of the transition from detention back to community, have to date been limited. A study on boys’ quality of life after discharge from secure residential care suggests that these adolescents were confronted with several difficulties, specifically in relation to social participation, family relations and finances [12]. However, they also experienced increased self-esteem and were more able to envision life goals than the control group of boys who were still admitted to the institution [12]. A study on girls’ quality of life in relation to mental health and offending behavior 6 months after discharge from a CI indicated that girls were most satisfied with their social relationships, but experienced difficulties in relation to their psychological health [13]. Our study contributes to the existing literature, as the studies that have been conducted in relation to the transition from youth detention centers to the community are either mainly quantitative (e.g. [2, 13]) or predominantly focused on the problems adolescents (may) experience following discharge from the institution (e.g. [14, 15]). Other qualitative studies focus exclusively on the period of ‘detention’ [16], or have a more narrow focus on either desistance from offending [17, 18] or resilience [19].

Throughout our study we focus on positive aspects and strengths during adolescents’ trajectories to better lives. This is not to ignore difficulties and the struggle adolescents may have gone through in this period, but rather to learn from what has been helpful to them, what is valuable and meaningful to them, and what inspires and motivates them for change. This study addresses the following research questions:

1. What is it like for adolescents to (re-)build personally valued lives after a court-mandated stay in a closed institution?
  - How did adolescents experience their stay in a closed institution?
  - Looking back, how do they make sense of their stay in the closed institution?
2. How did adolescents experience change and what has been supportive and motivating for them on their way to change?

## Methods

### Setting

In Flanders—the Dutch speaking part of Belgium—adolescents who exhibit antisocial and/or deviant behavior that may compromise their own or society’s safety, or adolescents who find themselves in an adverse living or educational situation, can be referred to a closed institution for mandatory care and treatment (CI). These institutions are in several ways comparable to youth detention centers in other countries, and have both a pedagogical and restrictive function [20]. Currently, the Flemish CIs are evolving from a pedagogical, social welfare model to a more risk management oriented model, in which adolescents are guided in their trajectories towards a better future by mitigating the risk of recidivism and enhancing their quality of life [21]. Placement in a CI is intended to get the adolescents “back on the right path”; to prevent recidivism through offering them a shelter, guidance and treatment; and to re-socialize and re-integrate the adolescents in preparation for their ‘return to society’ [20, 21]. Guidance in a CI is characterized by a highly confining and structured regime, in which the adolescents gradually receive more freedom and responsibilities. Furthermore, adolescents go to school on campus, and receive both a group based and individual educational, pedagogical and therapeutic program [21, 22]. In 2016, 914 adolescents, of which only 12.6% were girls, were placed in a CI for an average duration of 128 days [23]. The current study was conducted in the CI De Zande, one of the four Flemish CIs, which has a capacity for 100 boys and 54 girls [23]. In 2016, 193 boys and 115 girls were assigned to De Zande, with a mean length of stay of 148 days [23].

### Study design and procedure

The current qualitative study is part of a larger research project at Ghent University on detained adolescents’ quality of life and protective factors, and their relation to recidivism 6 months to a year after discharge from the CI. The project is a mixed methods study in which approximately 200 adolescents (boys and girls) are followed up by means of a four wave longitudinal research design: T0 in the first 3 weeks of their stay in the institution, T1 and T2 during their stay in the institution, and T3 when the adolescents have left the institution for at least 6 months. The following inclusion criteria were applied for adolescents’ initial participation in the study, and were assessed by the CI’s staff for each entering adolescent: (1) being sent to the CI for at least 1 month, (2) having sufficient knowledge of Dutch, and (3) having sufficient cognitive abilities to complete the questionnaires. Adolescents were eligible to participate in the qualitative study on condition that they were not residing in a CI again at the time of the interview.

The qualitative study is situated at T3, when the adolescents have been out of the institution for at least 6 months. At baseline measurement (T0), adolescents were asked for their willingness to participate in the following measurement moments. If they agreed, contact details were exchanged so that researchers were able to contact the participants again after they left the institution. At this last moment (T3), the questionnaires from T0 were repeated, and, for the first 25 adolescents who agreed to do so, an additional in-depth interview was conducted. All adolescents participated in the study on a voluntary basis, without any financial or material reward. Ethical approval for the study was obtained from the Ethics Committee of the Faculty of Psychology and Educational Sciences of Ghent University (E.C. decision: 2016/11).

### Sample

The study sample consists of both boys (*n* = 10) and girls (*n* = 15) who had been out of the institution for almost 8 months (*M* = 7.92; *SD* = 1.35; min. 6 months, max. 11 months). Eleven participants were referred to the CI because of an act defined as an offense (e.g. fighting, burglary, shoplifting, …), four participants because of an ‘alarming’ or adverse living situation (e.g. truancy, running away, prostitution, …), and 10 participants because of a combination of both. Nine out of the 25 participants were of non-Belgian origin (Moroccan, Tunisian or French). For 11 participants, it was their first stay in a CI, while 14 of them had already experienced one or several periods of detention. Participants’ age varied between 15 and 18 years old, with a mean age of 17.04 (*SD* = 0.889). At the time of the interview one participant was 15, six participants were 16, nine participants were 17 and nine participants were 18 years. Eight of the participants were living in an open institution at the time of the interview, seven of them were living with either one or both of their parents, four were living independently with some form of professional supervision and support, three of them were temporarily living with friends or distant relatives, and three participants were residing in a psychiatric institution. With regard to re-admissions to a CI; four participants had been re-assigned to the CI for a 2 week time-out program in the months between the moment they left the institution and the interview, one participant was sent back for 3 months, and one participant spent 4 months in adult prison.

### Interview

In-depth interviews were conducted with 25 adolescents who left the CI 6–12 months earlier. A topic list was used in order to systematically explore a number of themes (e.g. looking back at the period of detention and the subsequent months; reflecting on changes in life before and after staying in the CI; experienced strengths, sources of support and positive aspects in different life domains during and after the period of detention). This topic list could be adapted flexibly during the interview as participants were encouraged to speak as freely as possible. The interview location was agreed upon in consultation with the participants, and varied from the participants’ house or institution, to their school or day care center or a quiet public place. Participants were asked to do one-on-one interviews, but three of them felt more comfortable with a friend or relative nearby, so this choice was respected. All interviews have been conducted by the first author, who had already seen the participants at least one time—and most of them three times—during their stay in the CI. The average duration of the interviews was 73.03 min (range: 35 to 114 min). All interviews were audio-taped and transcribed verbatim, after which a thematic analysis was performed.

### Analysis

As a first step in the analysis, all interviews were read in depth several times and each individual story was reconstructed in a separate mind map in order to reveal the unique pathways and contributing elements for each participant. Based on the central themes that came to the fore in the mind maps, a thematic analysis was performed on all interviews using the software package NVIVO11, which enhances the transparency and efficiency of the coding process [24]. During this coding process, the initial “coding tree” was both expanded with relevant themes and subthemes, and some themes were re-organized, until a coding structure was reached which captured themes that hold for the majority of the participants; as well as singular, ideographic experiences, evaluations and appraisals. Smith [25] refers to this as “the balance of convergence and divergence” (p. 10) in which one strives to depict shared themes while at the same time looking for the particular meaning of this theme in each individual story. The results of our thematic analysis are presented by a schematic overview of the themes and subthemes that were identified. These themes are described and illustrated by means of participants’ quotes.

## Results

**Fig. 1:**
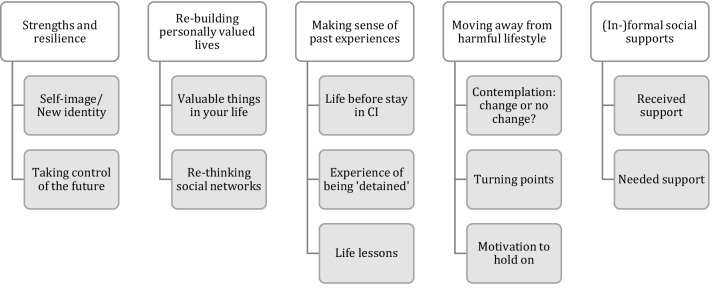
Themes and corresponding subthemes of adolescents’ experiences from the CI back to community

During the analysis process and based on the mind maps of all 25 interviews, five broad themes emerged out of the data: (1) strengths and resilience, (2) re-building personally valued lives, (3) making sense of past experiences, (4) moving away from a harmful lifestyle, and (5) (in)formal social supports. Each of these themes contains a number of subthemes (Fig. 1), which will be discussed in more detail below. The themes and subthemes show some overlap. This is connected to the nature of human narratives, which is complex, unstructured and full of paradoxes. Moreover, the dialectical process of the interview itself can re-structure and re-frame participants’ appraisal and sense making of their experiences.

### Experiencing strengths and resilience

This theme is closely related to the concept of ‘agency’ and can broadly be categorized in the subthemes: ‘self-image/new identity’; and ‘taking control of the future’.

### Self-image/new identity

Adolescents frequently mentioned low self-image or self-esteem when talking about the period before and during their stay in the CI, often accompanied by feeling ashamed of the things they had done in the past and the way others (used to) see them. However, adolescents who felt like they had succeeded in making some significant changes in their lives, argued that it made them feel better and proud of themselves, which in turn contributed to their motivation to hold on. In the same respect, adolescents emphasized the strength of important others (e.g. their parents, friends, a group worker he/she feels connected with, a teacher, …) noticing and appreciating these changes. For some, it was mostly through the eyes of others that they were able to start seeing themselves in a more positive way again. Consistent with experiencing improved self-esteem, participants sometimes tried to get rid of the old version of themselves by adopting a new identity, one in which they felt able to be proud of themselves.

*“People used to see me as a junkie, and they were right back then. But that is not who I am, not who I want to be. I am no longer a weirdo. My teacher said she sees me as a role model for some other students now. That makes me so proud. One of the first times I am actually proud of myself” (Adam, 17, living with parents)*

*“I was selected by the ‘Commissariat for Children’s Rights’ to be in the jury for a prize. We can say what is good and what goes wrong in childcare […] like a parliament, all very fancy, we even slept in a hotel. I told my story to some high*-*ranked people, one of them was fighting her tears, imagine that! I told everything I have gone through, all the pain and anger. My story moved her. She is a director or something like that, and now I am working with her, trying to find out how we can make things better” (Yasmine, 17, living in open institution)*

Adolescents in our study had often been—mostly involuntarily—the recipients of care and support in the past. Consequently, they enjoyed being able to switch the roles, and become the ones who gave support to others, who were able to—because of their own experiences—help others out. Wanting to protect younger siblings, or simply to be a good example for them was an important drive for some of them. Others indicated they do not want anyone to feel as bad or alone as they had been in the past.

*“Because of all that I have gone through in my life, I kind of feel like I have a special radar for people who are in trouble, I just feel it when I’m around them. I always try to help, either by listening or by distracting them from their problems. Everyone needs someone from time to time” (Sophia, 18, living independently)*

“*I just don’t want my little sister to make the same mistakes. From all these years, I have learnt when things can go wrong. I will to be there for her on these moments. I don’t want her to feel like she’s on her own.” (Lucas, 16, residing in psychiatric institution)*

### Taking control of the future

This theme is connected to the ‘self-image’ theme, as participants indicated that it was in relation to—and by virtue of—a growing self-confidence, that they started believing in their own capacities to create a better future. The decisiveness to manage their lives was very palpable in some participants’ stories. Furthermore, participants often stressed the importance of taking responsibility for their lives themselves, and not merely relying on others to improve their situation. This was also connected to recognizing and acknowledging their own share in mistakes from the past and drawing lessons from it for the future. Even though the individual responsibility for creating a better future was often stressed, some adolescents also referred to being able to ask help from others as a way of ensuring that everything went well.

*“A lot of people helped me and supported me in it [changing former lifestyle], and I am very grateful to them, but in the end, I was the one who had to make the switch in my mind, and then act accordingly, no one else could do that for me. […] I can count on them, and if things go wrong in the future, I will tell them. I’m not so stubborn anymore to think I can do it all by myself” (Isabella, 15, living in open institution)*

*“Every person must work on his own future. I am the only person who can ensure that everything goes well for me. I do not hope for a better future, because I just have to make it happen myself” (Oliver, 18, living with mother and brother)*

### Re-building personally valued lives

#### Valuable things in your life

This subtheme relates to inspiring and motivating elements in the adolescents’ life, and is related to the question “what gives direction and meaning to your life?”. Five of the adolescents—all of them Muslim—identified religion as the key element in their lives, helping them defy hard times and guiding them to make the right choices. Being able to experience and express their religion during their stay in the CI had been very helpful and strengthening for them.

*“My faith offered me some hope again, I had something good to focus on […] I have never been happy in my life. I could not believe that there is any God who would want that, so I thought of my stay [in the CI] as a chance from him to bring better things into my life” (Hannah, 17, living in open institution)*

While talking about what is valuable and inspiring in the adolescents’ lives, important others were frequently mentioned. Mostly, these important others were family members, such as parents, siblings or grandparents, with whom the adolescents experienced—or used to experience—a loving or caring relationship. Wanting these others to be proud of them and trust them (again) was a central theme in adolescents’ stories. Family members were mentioned most frequently (*n *= 12), but close friends (*n* = 8) and intimate partners (*n* = 7) also contributed significantly to adolescents’ willingness to change. Intimate partners were only mentioned by girls, while close friends were mostly referred to by the boys. Moreover, professional caregivers (*n* = 8) and school teachers (*n* = 6) can play a significant role in the adolescents’ life. Experiencing success at school, either by obtaining good grades, or by having teachers who believe in the adolescents and encourage them, contributed greatly to some adolescents’ sense of well-being.

*“She [former group worker] is the most important person in my life. She has always been there for me. I even got my very first birthday present from her. […] She comes to visit me from time to time […] I’m always looking forward to that, even though she nags at me when I’m behaving stupid”. (Charlotte, 17, living in a studio with professional support)*

*“My boyfriend, but also my teachers, they are the most important ones in my life […] They talk to me, they are interested in who I am, I can be a cheerful and enthusiastic girl when I am around them, not ‘that girl who lives in an institution” (Ella, 16, residing In psychiatric institution)*

*“I feel happy here [at school], they [teachers] don’t put too much pressure. Most of us are ‘problem children’, we all have our stories […] the atmosphere is good, we all respect one another. You don’t get punished for having a bad day. They talk to you, asking you what’s going on. That’s why it works for me… yell at me and I will do the opposite…” (Emily, 18, living with mother)*

When asked “what is important for you to feel good?”, adolescents mentioned a variety of themes. Some of these themes appear to be highly valued by most of the participants: (1) being surrounded by loved ones and experiencing pleasure with them; (2) experiencing freedom; and (3) themes related to ‘procedural justice’. The first aspect has been reported above. The second one, ‘experiencing freedom’, can be perceived on different levels: literally—as in not being locked up—and having the freedom to go when and where one wants to go; but also in a more figurative sense, as in being able to have your own thoughts and make your own choices, as well as to express yourself and to be able to show the ‘real’ you. Adolescents referred more often to freedom in this more figurative sense (freedom of mind) as one of the things they missed most during their stay in the CI, and which they highly valued in their current lives. As such, the freedom-theme is closely related to the third valued aspect: experiencing ‘procedural justice’. Several adolescents emphasized this theme as they had negative experiences with it in the past. Some examples of things that contributed to the perception of fair treatment are: being fully informed on one’s own trajectory, being listened to and having the opportunity to tell your version of a story, as well as being treated as a full-fledged discussion partner.

*“We all had our masks on [in CI], because if you really say or show what you think, you will probably get punished. It made me feel like a dog sometimes: be good and shut up. Here [current institution] I feel like I can say anything. That’s such a relief” (Yasmine, 17, living in open institution)*

*“They [juvenile judge and social worker] listened to me, but only because they are obliged to do so. They were not at all interested in what I was thinking, they had their mind made up in advance and that was it. It made me feel very powerless” (Nathan, 16, living with mother and sister)*

Participants’ goals were related to the life stage they were in and were connected to the desire of living more independent and autonomous lives. Finding a paid (weekend) job was the most frequently (*n* = 15) mentioned short-term goal, and being able to earn money was the predominant reason for the adolescents to want a job. Almost all adolescents (n = 18) were worried about their financial situation. Seven participants also stressed the importance of ‘having something useful to do’ and ‘not getting too bored’ (as they feared they would get in trouble then) as the main reason for wanting a job. Furthermore, some of them saw it as an opportunity to prove their good intentions to their parents or even the juvenile judge. Besides finding a job, other goals were related to school or education. For a large subgroup of the adolescents, this was an ambivalent goal, as they experienced turbulent school careers, often characterized by long periods of truancy or drop out. Some of them saw school as a finalized chapter in their lives, but most adolescents did hope to obtain a diploma or certificate 1 day in order to get a good job and an honest pay for it.

A striking observation during the interviews was that most participants, apart from some who had clear professional aspirations (e.g. working in restaurants, becoming a sports teacher or working in a day care nursery), seemingly did not really dare to dream or at least spoke very cautiously about their future aspirations. Most of them indicated they just hoped to be able to have a normal life and to be happy 1 day, and some of them expected that having a family of their own would contribute to that. As such, finding some form of inner peace, together with leading a more independent and autonomous life, seemed to be central themes in the adolescents’ current lives.

*“There is just too much going on […] I think the best thing I can hope for is that… I don’t know… One day I will have a normal life or something like that… That would be a lot already” (Oliver, 18, living with mother and brother)*

#### Re-thinking social networks

Throughout the adolescents’ stories, family and friends—and to a lesser extent intimate partners—played a very important role, either positive or negative. Mostly, they were a source of unconditional support, and the ones who brought joy into the adolescents’ lives. However, sometimes family members and friends were also jointly responsible for difficulties the adolescents experienced, which may have led them to take the decision of distancing themselves from these networks. The ambivalence concerning this theme, and the pain and doubt that went along with it, was very tangible in some adolescents’ accounts of their first weeks and months after leaving the CI. They felt torn between, on the one hand engaging in self-care by not seeing these persons any longer, but on the other hand missing them and the positive things they brought (e.g. joy, adventure, feeling important, …) into their lives. This led some adolescents to give up on their intention to stop seeing these others, while others persevered and actively focused on other persons in their lives or looked for new networks by joining a new sports club or going to another school.

*“I shut down all contact with her [mother]. She has never been good to me, but still, it hurts […] I try to surround myself with positive people […] I’m often with my aunt now, she is like a sister to me […] and I got back in touch with some girls from the youth movement I joined as a child” (Chloe, 17, living in open institution)*

*“[in the CI] I planned on not seeing my friends anymore, and I did in the beginning. But I don’t go to school, no job, I just played video games from morning until night. It drove my mom crazy. Not really an ideal life either, you know […] When they [friends] heard I was back, they came here to pick me up to go partying. Mom didn’t want me to go, but I did anyway. I felt happy again that night, like nothing had changed […] Life is just better with friends” (David, 18, living with mother)*

### Making sense of past experiences

Most adolescents perceived their stay in the CI as a drastic and stressful life event, using terminology as “my life before and after”. During their stories, they often tried to make sense of and seek for explanations for the things that happened in their lives and that led them to their current situations.

#### Looking back at life before detention

Adverse and traumatic childhood experiences (ACEs) were present in nearly all adolescents’ stories (20 out of the 25). Notwithstanding most adolescents’ difficult and harsh circumstances prior to their detention, they often referred to this period with a certain melancholy or nostalgia, describing it as ‘adventurous’, ‘fun’ and ‘making them feel alive’. Others described their lives before the CI mostly in negative terms as unhappy and sometimes desperate times.

*“I lived on the streets. I was often scared and lonely. At a certain point I was actively trying to get arrested so that I could get some rest and help” (Amy, 17, living in open institution)*

*“I often miss my former life [before stay in CI]. It was exciting and adventurous […] I felt more alive back then. but it also ruined me. I haven’t been to school since I was 14, I spent part of my teenage years behind bars, I screwed up with my family” (Aaron, 18, living independently)*

#### Experience of stay in the CI

Unsurprisingly, most adolescents did not like their stay in the CI, and feelings of being frustrated, lonely and powerless were often mentioned. However, adolescents also mentioned a variety of positive aspects connected to their stay in the CI; experiences, events or persons that offered comfort, encouraged them, motivated them and made them feel worthy. Seven adolescents described their stay in the CI as a shocking experience and consequently a real eye-opener; a starting point to turn their lives around. They talked about it as ‘an opportunity’ or ‘a chance being given to them’. Others perceived the CI as a sort of ‘moratorium’, a period in which they were taken away from their own environment, but in which nothing really changed, and afterwards everyone simply returned to his/her own life. A number of adolescents indicated that their stay in the CI was—at least in hindsight—a good opportunity for them to diminish or even stop using drugs.

*“It [not having drugs] was hard, but after a while, I started seeing things very clear again. It felt like the fog I used to be in was going away, and I could see a new me […] one who is alive, who is able to laugh and enjoy things […] It was like rediscovering myself” (Adam, 17, living with parents)*

Adolescents clearly differentiated between group workers and staff members who had been ‘good’ and ‘helpful’ to them and others who did not. Almost all adolescents had at least one group worker or staff member who was important for them, whom they experienced a trusting relationship with. The following key elements were emphasized as important aspects to perceive a relationship with the staff as positive: ‘experiencing warm and genuine care’, ‘being reasonable/being able to handle rules flexibly’, ‘getting trust’, ‘seeing the good in the adolescents’ and ‘being able to have fun’.

*“I felt closely connected to one of the group workers […] He was like me, ‘chill’. Not making a big deal of everything […] He made me push my boundaries during sports activities, but also on a more personal level” (Alex, 17, living in an open institution)*

*“They [two group workers in CI] cared for me in a parental and soft manner. I never expected that but it felt good. They made me feel important […] I still call them sometimes” (Eliza, 18, living with boyfriend)*

Furthermore, adolescents experienced support and pleasure by engaging in friendship relations with other adolescents in their group. Having friends in the institution seemed to contribute significantly to boys’ feelings of wellbeing. These friendships were described as rather superficial, mostly revolving around pleasure and a way to counteract boredom and isolation. For the girls, the friendship theme played out in a more ambivalent way. Eight of the girls indicated they kept distance from the group in the first weeks as they did not want to get involved with “those criminals or prostitutes”. However, almost all girls did engage in close friendships with others in their group after a while. Unlike for the boys, this seemed to induce high levels of distress for girls, with lots of gossiping and fights. Four girls, however, emphasize the close bond they experienced with other girls in their group as the most important element that helped them throughout their stay.

*“We [the girls] were always there for each other, helping each other out, you know, we have been through the same kind of stuff […] I had two very close friends in my group, we pulled each other up, they were like family to me” (Olivia, 17, living in open institution)*

Other elements that were perceived as helpful during some adolescents’ stay in the CI, were educational and sports activities, as they contributed to the feeling of ‘having something useful to do’ and ‘experiencing pleasure’. Whereas most adolescents complained on the amount of time they had to spend in their room, for some others these moments became valuable and it taught them new ways of organizing their free time (e.g. reading, writing in a diary, getting some rest, listening to music, making lists and plans for the future,…).

*“I learnt how to read in the CI. I knew how to do it from primary school but I have rarely been to school since then so I did not really […] But there, those first weeks, I was so bored that I started reading books […] it feels ridiculous to say but it changed my life. I spend every free hour at the library now” (Aaron, 18, living independently)*

Six adolescents were able to move to a more open group in the CI, in which they were gradually prepared for life outside the institution. Adolescents received more freedom in this group and also more responsibilities (e.g. having the chance to go on their own school or to have a job in the neighborhood of the institution). They talked about this as a very positive experience, as they had the feeling their group workers trusted and believed in them. The rules in this group were not as strict as in the other groups, which was highly valued by the adolescents. Moreover, being able to have contact with the outside world was perceived as very helpful.

#### Life lessons

Notwithstanding the fact that most adolescents perceived their stay in the CI as an unpleasant experience, most of them draw some important individual lessons from it. It made them re-think the choices they had been making in their lives up until then, it made them realize who and what was important in their lives and for some, it gave them hope for a better future. Being away from their own environments enabled some adolescents to look at their own lives from a different perspective, and to re-evaluate the people and activities in their lives. Furthermore, it gave them a clearer view of what they really wanted to achieve in their lives. For some adolescents however, the experience of being ‘detained’ was extremely frustrating, leading them to complete disinterest and even aversion of professional care.

*“It made me realize that I have to look after my own, that I should stand up for myself and not letting others determine my life and future” (Lucas, 16, residing in psychiatric institution)*

*“People change, at least I did… A lot of bad things happened in my life and at some points I was the one making it even more difficult. That makes me sad sometimes but the most important thing is that you learn from it […] When you’re in trouble, talk to people, when you’re feeling bad, talk to people. I used to hate all caregivers, but I know now that you just have to look for the good ones” (Amy, 17, living in open institution)*

*“It [stay in CI] definitely changed me. I still have nightmares sometimes. It made me anxious. I am never at ease anymore, because I know now that people can take away everything from you if they want to. At night, I make lists of everything I want to do, everything I want to achieve. It all has to happen here and now. I am only seventeen and I am looking for an apartment, I want a job, I want a partner and a child as soon as possible. Not later, but now, because I am afraid I won’t get the chance anymore […] I am not waiting any longer, if there is something I want, I go for it” (Charlotte, 17, living in a studio with professional support)*

*“The most valuable thing they [CI] have done for me, is giving me hope again. They made me believe that things can get better and that there are people out there who care about me” (Eliza, 18, living with boyfriend)*

### Moving away from a harmful lifestyle

At the time of the interview, most of the adolescents had already changed some aspects in their lives, or were currently trying to stop displaying harmful behavior (e.g. using drugs, stealing, getting into fights).

#### Contemplation: to change or not to change

Adolescents took divergent positions in relation to this theme. Furthermore, some adolescents switched from one position to another during the first weeks and months after ‘release’ from the CI. Most adolescents experienced some ambivalence in the decision on changing or not changing particular aspects of their lives. Some of the reasons or motivations for adolescents to change have already been discussed in the previous themes. The most important considerations or drives for change were: “to make important others proud (again)”; “because I have new responsibilities” (e.g. pregnancy, having to pay a house rent, having a job); and “for myself” (self-respect and growing self-confidence, improving health, for a better future). On the other hand, for those who choose not to change, or who ‘relapsed’ into old habits, the main considerations or reasons for this were: “reaching the age of legal majority/no more involvement of youth care”, “influence of (old) friends”, “financial considerations”, “being happy with one’s own life and corresponding lifestyle”, and “wanting to experience pleasure”.

*“I have changed a lot due to my relationship, but also just… you know, I have to do everything myself, living alone made me grow up. I have to pay my rent, have to clean my house, all those things. I don’t have time for the childish stuff anymore. You have to behave like a grown up, and not like a seven*-*year*-*old. That rebellious life is a bit over for me” (Jessica, 18, living independently)*

*“I try not to do it [stealing] anymore, because if I get caught I would be too ashamed to ever look my parents in the eyes again […] but sometimes I have a girl, you want to have a drink, take her on a date… You need money for that…” (Nathan, 16, living with mother and sister)*

*“It was the best time of my life, the worst because we had nothing, but the best because we did whatever we wanted to do, we did not care about anything or anyone, just having fun, all day, all night […] I could be me, just me. Now people expect me to become a new me, a boring version of myself, but what’s in it for me?” (Dylan, 18, living with relative)*

#### Turning points

This is closely related to the contemplation-theme. For some adolescents—describing their stay in the CI as a life changing event—the mere fact of being sent there can be seen as a turning point. For others, turning points were linked to people rather than to specific moments in time. Five adolescents designated their current boyfriend or girlfriend as the ones who were responsible for and motivated them in their change process. Others were mostly prepared to make some changes because they wanted their parents and siblings to be proud of them, and because they wanted to become proud of themselves again. Friends and peers could both play a supportive and encouraging role for adolescents in changing or maintaining their new lifestyle. However, some adolescents’ stories showed that friends could trigger relapses in old habits as well. Building up new networks appeared to be a very powerful—yet hard to realize—hook for change. These networks were sometimes found by joining a new sports club, or for some adolescents by moving to a new school or a new (open) institution. Having people in their life was a first step, but an even more compelling aspect for the adolescents was that these people genuinely cared about them, and made them feel worthy and important. Some adolescents indicated ‘getting a (new) chance’ as a hook for change, e.g., getting in contact with and apologizing to their victims, getting a job, being re-admitted to their old school, having the chance to live independently (mostly with professional support), getting financial support…. Furthermore, being able to address faults from the past, and to be forgiven or be seen differently by others was an important turning point in some adolescents’ lives.

*“I am not proud of what I have done, but I am not ashamed either. I have done my sentence and I learned from it […] I don’t want to keep living in the past […] I got the chance to come here, to go to school again, I am doing good, my teachers like me and I get along very well with my group workers. Why would I want to ruin that?” (Chloe, 17, living in open institution)*

### (In-)formal supports

#### Received support

Adolescents’ stories showed that both formal and informal networks can play a significant supportive role in their lives. Adolescents experienced support from their family, intimate partner, friends and peer group, but also from school, teachers and professional caregivers—provided that the relationship was perceived as warm and sincere. Professional home based counselling following the period of detention was an ambivalent theme for a number of adolescents, because of the mandatory nature of this care. Notwithstanding adolescents indicated that they needed some form of support during this period, the received care was sometimes perceived as “too much, too invasive and too controlling”. For some, this made them feel as if they were not trusted and as if they were still being punished for the things they had done.

*“When I am having a dispute or trouble with my mom, I can call her [home based counsellor], I can talk to her, that calms me down […] She is young, it is like talking to another youngster, but still it is different, because you don’t discuss problems with your friends […] I have to see her three times in a week, so I will be relieved when it stops, because there are times when I don’t have anything to say to her because everything is just normal. I would rather spend my time with my friends or girlfriend then” (Nathan, 16, living with mother and sister)*

#### Needed support

Most adolescents received some kind of support from their own network of friends and family. However, four adolescents indicated they have no social network to rely on, only the professional caregivers in their institution. While professional support, either in the form of residential care or home based counselling, was perceived as very supportive and helpful by about half of the adolescents, others referred to some difficulties connected to this. Some adolescents had the feeling their professional caregivers were preoccupied with providing emotional support, while at some points in their trajectories, adolescents mainly needed practical and financial support. They felt left out in the cold, and felt unable to tackle these challenges on their own. Furthermore, adolescents had the feeling that the structured way in which professional care was organized (e.g. having to go there at fixed times or someone coming to your house several times a week) was not an adequate answer to their support needs at that time, and was consequently sometimes perceived as a waste of time. This was connected to some adolescents’ frustration of not being taken seriously and not being listened to, which consequently led them to feeling powerless and unable to direct their own life.

*“I have considered going to one [psychologist], because it’s been a lot and there are days when I feel like I cannot do this on my own. But most days I am feeling ok and I don’t feel like talking about my past. But it doesn’t work like that. You have to make an appointment and then you have to go, no matter how you feel that day. If you have a good day, it might spoil the rest of your day, do you understand? I just need someone for those days when I feel miserable and when I can’t manage to get out of my bed, but you cannot expect these people to work like that” (Sophia, 18, living independently)*

*“The only thing they have to do is listen to us, not treating us as if we are children or criminals or whatsoever, just talk to me, you know, like you would talk to a normal person. Just come to my house or have a drink with me, then you will maybe get to know me. My social worker invites me in her office two times a year, we sit there in this crazy white room and she is convinced she knows me and my family so good, that she can say what has to happen to us in the next year. I get very upset by that, because it feels as if they have taken away a large part of my childhood, and for what?” (Irene, 17, living with mother and sister)*

## Discussion

In this section, we first formulate an answer to our research questions, followed by a more global discussion and reflection on the results of our study. Furthermore, we discuss the strengths and limitations of this study, as well as its implications for research and practice.

### What is it like for adolescents to (re-)build personally valued lives after a court-mandated stay in a closed institution?

Adolescents experienced their return to ‘regular life’ in different ways, especially because—at least for some of them—several aspects of their lives had drastically changed after their stay in the CI (e.g. being admitted to a new open institution, going back to school for the first time in years, not using drugs anymore, …). Some adolescents perceived these changes as positive and were predominantly enjoying their regained freedom and the new opportunities it brought them. For others, they felt lost and had the feeling they ‘fell into a black hole’. Examples of this are: a girl who is not hanging out with her former deviant peer group anymore, but who has no other friends either; a boy who stopped selling drugs, but has no job or income; or a boy who quit doing burglaries, but misses the tension and adventure it brought into his life. According to the GLM [26], one could say that these adolescents’ trajectories were mainly guided by avoidance goals, with only limited scope for approach goals. This can be explained by the fact that some of these adolescents omitted or ceased several aspects of their former ‘socially unacceptable behavior’, often under pressure from others such as their parents, caregivers or the juvenile judge, but no—or only limited—positive replacements have taken place. As a consequence, they did not feel satisfied with their current lives, and were balancing and bouncing back and forth between holding on to this new lifestyle, or falling back into old behavior. This might imply that moving forward in the direction of a better life unfolds through a pattern in which adolescents first have to go through a difficult period—for instance by feeling a sense of loss in relation to their older life—after which they become able to reconstruct their lives again and through that return to a good quality of life. A similar pattern was also seen in a study with girls recovering from anorexia nervosa [27] and is consistent with Cummins’ subjective wellbeing homeostasis theory [28].

As placement in the CI induced—to a greater or lesser extent—discontinuity in adolescents’ lives [29], most adolescents seemed to be looking for some new balance in their life, and highly emphasized the role of “important others” in this. Experiencing trusting relationships with people who are supportive, genuinely interested and committed, and who believe in them was deemed important in adolescents’ accounts of what made them value their lives. This corresponds with a study conducted with adolescents in residential youth care, in which ‘interpersonal relations’ (i.e. having supportive and reliable friends and family) was designated by these adolescents as the most important domain for being able to experience a good quality of life [30]. Alongside support, adolescents also often experienced high levels of pressure from their environment (e.g. parents being overly controlling, very strict rules in the institution or frequent mandatory contact with home based counsellors) and they felt like having to prove themselves constantly. This ‘pressure to perform’ was also found in a study of a different target group (in this case mentally ill offenders) in secure forensic settings [31] so this might be an inherent tension in mandatory treatment. While some adolescents perceived this pressure as a motivation to ‘do good’, others perceived it as too much and too stifling, leading them to either disinterest, rebellious behavior and/or disengagement from professional caregivers.

#### How did adolescents experience their stay in a closed institution?

Adolescents made frequent references to feeling frustrated, lonely and powerless, especially in the first days and weeks of their stay in the CI. This is consistent with findings of Van Damme and colleagues [32] who found a clear drop in the quality of life of girls after admission to the CI, and is consistent with other qualitative studies in which this was found to be a highly stressful experience, as adolescents were cut off from their social networks and daily lives, and were limited in their autonomy and self-determination [16, 33]. Adolescents rarely referred to specific treatment-related aspects when talking about what contributed to or influenced their trajectories in a positive way. The things that mattered most during their stay appear to be situated at the level of warm human contact: feeling closely connected to and supported by staff members (mostly group workers) and/or other adolescents, and being able to experience pleasure with them. This association between perceived social climate and therapeutic relationships, and satisfaction with forensic services has also been emphasized in a study of Bressington and colleagues [34]. Our results show that being treated with respect and authentic care, as well as being treated in a reasonable and fair way, highly contributed to adolescents’ sense of wellbeing during their stay. This resonates with findings on ‘procedural justice’ in other studies [35] and refers to aspects such as being fully informed of one’s own trajectory and prospects, as well as being listened to and having a say in decisions. This is also compatible with a recent study on adolescents’ experiences of repression in residential youth care, which decrease if their autonomy is respected and treatment is perceived as more personally meaningful [36].

#### Looking back, how do adolescents make sense of their stay in the closed institution in relation to their current lives?

For some adolescents placement in the CI was perceived as a shocking and eye-opening experience, leading them to the decision of bringing about some important changes in their lives. Looking back, others see their stay in the CI as an opportunity—albeit an unpleasant and forced one—to diminish or even quit using drugs. For a number of adolescents, their time in the CI was important as it gave them hope again for a new start and a better future, and it strengthened self-confidence as they acquired some new coping strategies. However, some adolescents also saw their stay in the CI as a waste of time, in which nothing changed, and they just went back to their old lives afterwards.

### How did adolescents experience change and what has been supportive and motivating for them on their way to change?

In most adolescents’ stories there was a tangible tension between, on the one hand wanting to change, and on the other hand missing—some aspects of—their former lifestyle. This was mainly the case with regard to ‘experiencing pleasure, joy and adventure’ in their lives. Furthermore, having a clear vision of what one wants to do, or achieve, in the future (e.g. graduating, having a job, living more independently), seemed to be an important drive for adolescents to hold on to a new, more prosocial lifestyle. This is in line with recent findings on the role of envisioning prosocial future selves in the way to desistance [37]. Experiencing success in one way or another, which is noticed and appreciated by important others, provided adolescents with the self-confidence needed to tackle their future, which has been referred to as the looking-glass self-concept, and is related to the importance of ‘being welcomed back into society’ [38]. Furthermore, certain life events or experiences played out as ‘hooks for change’ [18, 39] for the adolescents (e.g. expecting a baby, finding a job, a new boyfriend or girlfriend, …). However, some adolescents seemed to be missing the social or economic capital needed to be able to move towards better lives. Being surrounded by a solid and caring network of friends, relatives or professional caregivers—or at the very least one important other—in combination with having access to basic resources can be seen as a minimum set of elements in adolescents’ motivation and perseverance to change.

A global finding, when looking over the 25 stories, is that ‘change’ can be perceived on a continuum ranging from ‘no change at all’ to ‘a lot of change’, in which periods of relapse into old ‘socially unacceptable’ behavior (e.g. drug use, criminal offenses, truancy, running away from home, …) frequently occured, often following a certain setback such as a break-up, an argument at home, or a period of unemployment. This is in line with the process-driven and on-going nature of desistance, as described by—amongst others—Farrall et al. [40] and Hunter and Farrall [37]. A similar movement can also be seen in relation to boys’ [12] and girls’ quality of life [32] during and after stay in a CI. Furthermore, when taking a closer look at the mind maps that were made of each individual participant’s story, we see that both intertwined aspects connected to leading a good life—‘feeling good’ and ‘behaving good’—were combined in different ways and that, at least for a subgroup of the adolescents, one did not necessarily co-occur with the other. In other words, leading a life that is perceived as personally meaningful, does not imply that this life aligns with society’s normative expectations and standards, and vice versa. Taking account of this observation—however explorative—we concur with the GLM’s basic assumptions [4, 7, 26] on the importance of combining and integrating both aspects in rehabilitation efforts: supporting people in getting away from a harmful lifestyle by helping them in the process of discovering what is important and valuable to them, and guiding them in achieving this valued life. Hence, treatment efforts should be directed on enhancing adolescents’ quality of life in those life domains that matter most to them. Further research that unravels the specific and possible interactions between the normative and personal aspect of leading a ‘good life’ could be important, as it can broaden our knowledge and understanding of different pathways to leading better lives, and the drives and motives that are central in these pathways.

Many of the themes that were found to be important for the adolescents in our study are in some ways prototypical for and might—to a greater or lesser extent—apply to all adolescents (e.g. importance of experiencing pleasure and adventure or striving for more autonomy). However, there are also important differences, for instance with regard to the structural barriers one has to overcome in life (see also Giordano et al. [18]), and associated with that experiencing a more limited discretionary field to explore and experiment with different roles on the road to growing up to become ‘responsible citizens’. Almost all adolescents in our study made reference to one or more adverse or traumatic childhood experiences, and most of them had already been living in institutions for at least a couple of years. Furthermore, a large subgroup of the adolescents worried about their financial situation and (future) housing. This is consistent with findings on the high prevalence of adverse childhood experiences in juvenile offenders’ lives [41–43], and supports the need for further research on the relationship between experiencing trauma and offending behavior, as well as on trauma-informed interventions [44].

Even though most adolescents grew up in challenging and difficult situations, some of them somehow appeared to succeed in leading better lives. This might lead to the presumption that some adolescents are more resilient than others, as well as to the structure-agency debate that has been well described in the desistance literature (e.g. [40, 45, 46]. It might be that for those adolescents, at some points along their way, more ‘hooks for change’ [described by Giordano and colleagues [18] as “potentially prosocial features of the environment as catalysts, change agents, causes or turning points” (p. 1000)] have been available than for others. A central aspect in “hooks for change” is people’s openness to these hooks and their agency for ‘grasping’ them [18]. However, agency can only be understood in relation to having choices and opportunities in life and in relation to having the capabilities and capacities to exercise it [18, 39]. As such, the ability to exercise agency is closely related to, and dependent on the adolescents’ own possibilities and social supports in overcoming structural barriers that exclude them from these choices, which has also been described by Gray [45]. For some adolescents in our sample, these barriers were at the moment of the interview simply too great to overcome, and they did not (yet) receive—or had no access to—the help or support they needed in doing this. Similar findings are reported in a follow-up study by Harder and colleagues [14]. This is an important consideration for both policy makers and practitioners in rehabilitative treatment programs. One cannot expect adolescents to ‘work on themselves’ and their goals, while their current circumstances are constraining this, for example because of not having access to decent housing or financial resources or because of a drug addiction. This aligns with the GLM’s emphasis on tackling the obstacles that restrain people from living a life that is perceived as personally valuable [26]; and with Colman and Vander Laenen [47] who found in a sample of drug-using offenders that, before desistance can occur, offenders see recovery from drug use as the first important step. This might also hold to recovery in a broader sense, as in overcoming mental health problems, but also on a more societal level, as surmounting the consequences of social, cultural or economic exclusion (see also Giordano and colleagues [18]).

## Strengths and limitations of the study

The present study contributes to the existing strengths-based literature as it highlights—starting from adolescents’ own perceptions and experiences—the strengths, positive aspects and motivating elements on their way to ‘better’ lives. As such, we combine the focus of desistance research on socially desirable outcomes, with a more client-centered perspective, focusing on quality of life.

However, there are several limitations; one of them being the heterogeneity of our study sample. Adolescents can be referred to a CI because they have committed criminal offenses, but also because of an adverse living situation. We included both groups in our study. Merely seen from a desistance point of view, this would be a remarkable and even unjustifiable thing to do as the second group has not been placed because of criminal offenses. However, we operationalized change in a broader and more holistic sense, as in moving away from a harmful lifestyle (for themselves or for others) and towards ‘growth and change for the better’.

While we explicitly discussed our focus on positive aspects and strengths with the participants at the start of every interview, negative or adverse experiences were often discussed during the interviews. One explanation could be that people tend to remember negative events or feelings more vividly than positive ones, or that the participants are better used to talking about problems than about things that are going well. Above all, this might be indicative of the ‘harsh and bumpy road’ these adolescents have gone through, or are still going through. When reading and interpreting the results of our thematic analysis, one should keep in mind that we mainly focused on the positive elements in adolescents’ narratives. However, difficulties and struggles adolescents experience(d) are acknowledged and taken into consideration in our discussion and reflection in terms of the relation between adolescents’ perceived quality of life and leading a ‘normative good life’.

We exclusively focused and relied on information from the adolescents themselves, as we wanted to learn from their stories and perspectives, and were mainly interested in their lived experiences. This implies that the information (e.g. on current ‘deviant’ behavior) has not been checked in any official records. As such, we cannot identify the impact of social desirability on the adolescents’ answers and stories. However, a relationship of trust with the interviewer was established to some extent for all participants, as the interviewer had already talked to them at least one time—and in most cases three times—during their stay in the CI.

The five broad themes that have been presented in our results section, are based on a thematic analysis that was performed on the data. Although this thematic analysis was helpful in identifying, analyzing and reporting certain patterns [48] in the adolescents’ stories, it also left us with a more fragmented image of the adolescents’ narratives. The cohesion between different themes and the way in which they interact and play out differently in each individual story sometimes got lost as a consequence of the process of ‘cutting and pasting’ themes in a broader structure. We do see this cohesion when looking at the mind maps we made of each individual story. Whereas our current study provides an overview of the relevant themes on a group-level, it would also be interesting to take a closer look at how these themes play out on an individual level. Based on a detailed analysis and understanding of particularities as well as differences, further research can inform us about how to rethink and accommodate treatment and interventions to the specific needs of these adolescents.

## Conclusion

Our study aimed to investigate positive aspects and strengths in formerly detained adolescents’ trajectories to better lives. We found that most adolescents were on their way to finding a new balance in their lives, however, for some of them this was still very fragile. Positive goal-directedness, still being able to experience pleasure and joy in one’s life, and feeling closely connected to and supported by someone who believes in them, supports them and genuinely cares for them, appeared to be highly important elements for the adolescents in our sample. We argue for strengths-based approaches in forensic treatment with a focus on enhancing adolescents’ quality of life by targeting those life domains that matter most to them, as these can foster hope and motivation for a better future again.

## Abbreviations

GLM: Good Lives Model of Offender Rehabilitation
CI: closed institution for mandatory care and treatment
ACE: adverse childhood experience

## Footnotes

1 We sometimes use the terms ‘detained adolescents’ and ‘detention’ throughout this manuscript, in consideration of comparability in an international context. Our study was conducted in a CI in Belgium, which is not completely the same as a youth detention center, as both adolescents who have committed offenses as well as adolescents who find themselves in an adverse living—or educational situation can be referred there by the juvenile judge. However, due to the closed nature of these institutions—both in infrastructure and in regime—and due to the mandatory character of the provided care, CI’s are in several ways comparable to youth detention centers in other countries.
